# The complete mitochondrial genome and phylogenetic position of the Scalpel sawtail *Prionurus scalprum* Valenciennes, 1835 (Acanthuriformes: Acanthuridae) from Jeju Island, Korea

**DOI:** 10.1080/23802359.2022.2109434

**Published:** 2022-08-22

**Authors:** Jae-Hoon Kim, Hyo-Won Kim, Jin-Woo Park, Seung-Chul Ji, Jeong-Hyeon Cho, Jae-Koo Noh, Dae-Jung Kim, Jung-Hyun Kim

**Affiliations:** a Jeju Fisheries Research Institute, National Institute of Fisheries Science, Jeju, Republic of Korea; b South Sea Fisheries Research Institute, National Institute of Fisheries Science, Yeosu, Republic of Korea

**Keywords:** Scalpel sawtail, *Prionurus scalprum*, complete mitochondrial genome

## Abstract

Although the mitochondrial genomes of most Acanthuriformes fish have been sequenced, this has not been done for Prionurinae fish yet. The Scalpel sawtail (*Prionurus scalprum* Valenciennes, 1835) found in all tropical and sub-tropical seas, is an important link between primary producers and higher trophic levels. In this study, the complete mitochondrial genome of the Scalpel sawtail, *Prionurus scalprum* Valenciennes, 1835 (accession number: OK323243) was sequenced. The complete mitochondrial genome included 13 protein-coding genes (PCGs), 22 transfer RNA (tRNA) genes, two ribosomal RNA (rRNA) genes, and a control region; the total length was 16,522 bp. The nucleotide composition of the genome was as follows: A, 28.46%; T, 27.62%; G, 16.46%; and C, 27.46%. All genes were encoded on the H-strand, except for the eight tRNA genes and NADH dehydrogenase subunit (*ND6*). A phylogenetic tree was constructed using the neighbor-joining (NJ) method and the phylogenetic position of the Scalpel sawtail was established. This provides useful data for future research on Acanthuridae fish.

The Scalpel sawtail (*Prionurus scalprum* Valenciennes, 1835) belongs to the Acanthuridae family in the order Acanthuriformes. This fish inhabits coral reefs in all tropical and sub-tropical seas at a depth of 2–20 m. Most are herbivores and provide important ecosystem functions for coral reefs by maintaining algal turf biomass, and serving as an important link between primary producers and higher trophic levels consumers (Marshell and Mumby 2015). The mitochondrial genomes of most Acanthuriformes fish have been sequenced, except for Prionurinae fish (Yamanoue et al. 2007; Devadhasan et al. 2016; Huang et al. 2017; Li et al. 2020a, 2020b; Yang et al. 2020). Here, we report the sequenced mitochondrial genome of *P. scalprum* for the first time.

Sample was distinguished based on the description of Masuda (1984) and the Aquatic Life Resources Information Center affiliated with the National Institute of Fisheries Science (NIFS). We analyzed the complete mitochondrial genome of the Scalpel sawtail, which was sequenced at the Jeju Fisheries Research Institute, NIFS, Jeju Island, Republic of Korea (33°16′17.4″N, 126°40′48.4″E), and deposited in the specimen room of the Biotechnology Research Division, NIFS, Busan, South Korea (accession no. NFRDI-FI-TS-0061797, Eun-Soo Noh, laperm@korea.kr). Samples for analysis were collected from Scalpel sawtail held for species conservation analysis. Library was prepared with the MGIEasy DNA library prep kit (MGI, Shenzhen, China) according to the manufacturer’s instructions. Briefly, after size-selection of fragmented gDNA using AMPure XP magnetic beads, the fragmented gDNA was end-repaired and A-tailed at 37 °C for 30 min and 65 °C for 15 min. An indexing adapter was ligated to the ends of the DNA fragments at 23 °C for 60 min. After cleanup of adapter-ligated DNA, PCR was performed to enrich the DNA fragments with the adapter molecules. Thermocycling conditions were as follows: 95 °C for 3 min; seven cycles of 98 °C for 20 s, 60 °C for 15 s, and 72 °C for 30 s; and a final extension at 72 °C for 10 min. The double stranded library was quantified using the QauntiFluor ONE dsDNA System (Promega, Madison, WI). The library was circularized at 37 °C for 30 min and then digested at 37 °C for 30 min, followed by cleanup of the circularized product. To prepare DNA nanoballs (DNBs), the library was incubated at 30 °C for 25 min using the DNB enzyme. Finally, the library was quantified using the QuantiFluor ssDNA System (Promega, Madison, WI). The prepared DNBs were sequenced on the MGIseq system (MGI, Shenzhen, China) with 150 bp paired-end reads. The complete mitochondrial genome of *P. scalprum* was assembled by mapping clean reads against the reference sequence, from *Prionurus laticlavius* (MN703418). Annotations were performed with the MITOS web server (Bernt et al. 2013) and corrected manually. In addition to MITOS, the tRNAscan-SE v. 2.0 web server (Lowe and Eddy 1997) was used to identify the transfer RNA (tRNA)-coding regions. The resulting mitochondrial genome was visualized in OGDRAW v. 1.3.1 (Greiner et al. 2019). The complete mitochondrial genome of the Scalpel sawtail was 16,522 bp long, with 13 protein-coding genes (PCGs), two ribosomal RNA (rRNA) genes, 22 tRNA genes, and a control region (*D-loop*). Encoded genes were similar to those of other Acanthuriformes (Li et al. 2020a, 2020b; Yang et al. 2020) and Perciformes (Kim et al. 2018; Park et al. 2020) fish. The length of the 13 PCGs was 11,429 bp; making up 69.17% of the mitochondrial genome. The nucleotide composition of the mitochondrial genome was as follows: A, 28.46%; T, 27.62%; G, 16.46%; and C, 27.46%. The G + C content (43.92%) was lower than the A + T content (56.08%). All the genes were located on the H-strand except for eight tRNA genes (tRNA-*Pro*, tRNA-*Glu*, tRNA-*Ser*, tRNA-*Tyr*, tRNA-*Cys*, tRNA-*Asn*, tRNA-*Ala*, tRNA-*Gln*) and NADH dehydrogenase subunit (*ND6*). The 22 tRNA genes ranged from 67 bp to 75 bp in length. The start codon for most of PCGs was ATG, but *COX1* started with GTG. Seven of the PCGs (*ND1*, *COX1*, *ATP8*, *ATP6*, *ND4L*, *ND5*, *ND6*) stopped with TAA, while *ND2*, *COX2*, *COX3*, *ND3*, *ND4*, *CYTB* stopped with an incomplete stop codon (T or TA). The *12S rRNA* was 948 bp long and was located between tRNA-*Phe* and tRNA-*Val*. The *16S rRNA* was 1693 bp long and was located between tRNA-*Val* and tRNA-*Leu*. The control region (*D-loop*) was 851 bp long and was located between tRNA-*Pro* and tRNA-*Phe*. We constructed a phylogenetic tree using the NJ method based on the complete mitochondrial genome sequence (Figure 1). This result provides potentially useful data for future studies on Acanthuridae fish.

**Figure 1.**
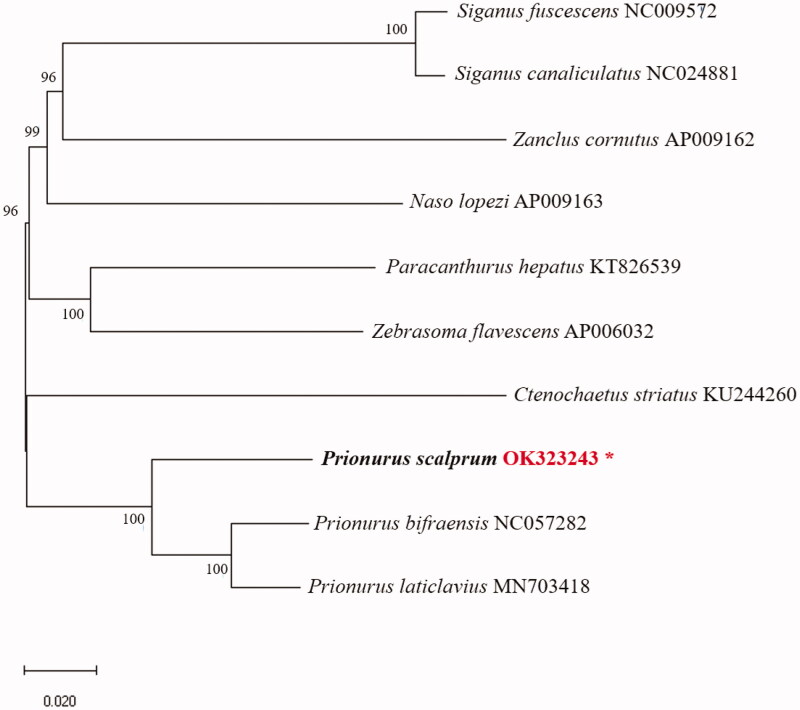
Phylogenetic position of the Scalpel sawtail *Prionurus scalprum* from Jeju Island, Korea.

## Data Availability

The data that support the findings of this study are openly available in GenBank at https://www.ncbi.nim.nih.gov/genbank, accession number OK323243. The associated Bio Sample, BioProject, and SRA numbers are SAMN24907634, PRJNA796720, and SRR17594202, respectively.
